# The STORY MINE Digital Recovery Narrative Intervention for Personal Recovery in Mental Health Care: Pilot Randomized Controlled Trial

**DOI:** 10.2196/83948

**Published:** 2026-09-30

**Authors:** Mitch N Lases, Jojanneke Bruins, Ingrid DC van Balkom, Stynke Castelein

**Affiliations:** 1 Department of Clinical Psychology and Experimental Psychopathology, University of Groningen, Faculty of Behavioural and Social Sciences, Groningen, The Netherlands; 2 Lentis Research, Lentis Psychiatric Institute, Hereweg 80, Groningen, 9725 AG, The Netherlands, 31 610654261; 3 Department of Psychiatry, Rob Giel Research Centre, University of Groningen, University Medical Centre Groningen, Groningen, The Netherlands; 4 Research Department, Trajectum, Zwolle, The Netherlands

**Keywords:** mental health, recovery narratives, ISMI, transdiagnostic, digital intervention, eHealth, minddistrict, podcast, Internalized Stigma of Mental Illness Scale

## Abstract

**Background:**

Hope and confidence support personal recovery by helping individuals with mental illness to rediscover meaning, enhance social connections, and build fulfilling lives. Digital recovery narrative interventions may help stimulate these recovery processes by providing access to hopeful lived-experience stories.

**Objective:**

This pilot feasibility randomized controlled trial evaluated the feasibility, acceptability, and exploratory outcome signals of STORY MINE, a novel digital intervention based on hopeful recovery narratives, to support personal recovery among adult service users receiving specialized mental health care at a Dutch psychiatric institute.

**Methods:**

Adult service users receiving outpatient specialized mental health care were recruited through clinician referral and self-enrollment. Participants (N=28) were randomized (1:1) to either intervention (STORY MINE access for 12 wk alongside care as usual) or control (care as usual only). Feasibility and acceptability were assessed via platform usage data, evaluation questions embedded within the intervention, and postintervention feedback. Quantitative outcomes were assessed at baseline and postintervention using validated self-report questionnaires on mental health confidence, hope, self-stigma, and personal recovery. Descriptive statistics and exploratory effect sizes with 95% CIs were calculated. Qualitative feedback was analyzed thematically to explore user experiences and perceived impact.

**Results:**

All participants (N=28) completed self-report questionnaires, while 13 of 14 participants in the intervention group provided evaluation feedback. Feasibility and acceptability were rated positively (mean 7.7, SD 2.52). Engagement was low, with a mean of 2.77 (SD 1.49) active login days per participant over 12 weeks, with some participants reporting minor technical barriers to use. Participants reported mixed perceived recognition (identification with narratives) and relevance (perceived applicability), but qualitative findings indicated perceived benefits including increased meaning-making, inspiration, and connectedness to recovery narratives. Some reported STORY MINE contributed to their recovery process (eg, narratives provided practical tips and evoked meaning-making and more positive self-evaluation). However, mixed emotional responses were reported; one participant reported narratives triggered distressing memories. Exploratory analyses showed small negative effect sizes for mental health confidence (Cohen *d*=−0.32, 95% CI −1.07 to 0.42), hope (Cohen *d*=−0.45, 95% CI −1.20 to 0.30), and self-stigma (Cohen *d*=−0.33, 95% CI −1.08 to 0.42), and a medium negative effect size for personal recovery (Cohen *d*=−0.53, 95% CI −1.28 to 0.22). These effect estimates decreased after excluding 2 participants experiencing an acute mental health crisis during the study period.

**Conclusions:**

STORY MINE appears feasible and acceptable as a supplement to specialized mental health care, although low engagement and mixed emotional responses highlight the need for refinement, particularly to improve user engagement, emotional safety, perceived relevance, and accessibility of the intervention. Findings should be interpreted cautiously given the pilot design and small sample size. A fully powered randomized controlled trial is warranted to evaluate effectiveness.

## Introduction

Hope and confidence [1,2] may be powerful factors driving personal recovery, enabling people with mental illness to reclaim meaning, strengthen social connections, and build a sustainable future. Initiatives promoting personal recovery have mostly emerged and expanded outside formal mental health care and include peer support networks [3-5] and recovery colleges [6-9].

There is increasing attention for the importance of lived experience-based knowledge in mental health care [10,11]. Several studies indicate that exposure to experiential knowledge, shared through recovery narratives, could help counter recovery barriers such as low confidence, hopelessness, and (internalized) stigma and have the potential to improve outcomes such as quality of life and hope in people with mental health problems [12-18].

Recovery narratives offer the potential to induce hope through experiencing connection [12], therewith opening the perspective for an alternative meaningful life story. Identification with personal experiences and developing an alternative, more agentic life story are also seen as the most important elements for mental health in effective life-review and narrative therapy [19-23] approaches. Recovery-oriented practices and the use of recovery narratives are increasingly recognized as complementary components to clinical treatment by offering concrete learning examples of how progress in all recovery domains can be reached in one’s prospective life. They offer accessible resources that may bridge gaps in care, particularly in the domains of personal and societal recovery [5,24,25].

Exposure to recovery narratives has the potential for both positive and negative effects [15], depending on the content and characteristics of the narratives and on how, when, and to whom they are presented [26]. Positive effects can be promoted by hopeful narratives containing practical content and displaying a recovery stage not too distant from the recipient (ie, not fully recovered), thus making the recovery goal more realistic to achieve [12,27]. The opportunity for positive change in recipients is higher when people are engaged with a narrative, usually when feeling connected because of similar narrator characteristics or recognizable experiences that transcend diagnostic categories [12]. Negative effects may occur when narratives evoke feelings of inadequacy, pessimism, or disconnection. For instance, a narrative portraying rapid or full recovery could feel out of reach for recipients, causing them to doubt their own progress or feel they are falling short in comparison [15,27].

However, existing recovery narrative interventions often rely on preexisting or opportunistically collected narratives, with limited control over narrative content, structure, or alignment with recovery-promoting mechanisms [14,16,17]. One example of such an intervention is the Narrative Experiences Online (NEON) program, which evaluated a digital intervention providing access to a large, curated collection of recorded mental health recovery narratives for people experiencing mental health problems [18]. While digital interventions such as the NEON trials have demonstrated feasibility and potential effectiveness, challenges remain regarding user engagement, personalization, and the safe delivery of narratives within vulnerable populations [13,18].

Although recovery narratives are nowadays publicly available in various places, collections are often curated based on existing narratives or shared at random, without knowing whether the content actually contributes to the recovery process of the recipient. Furthermore, there is a lack of interventions that deliberately curate and design narrative collections based on theoretically grounded “active ingredients” that are expected to promote recovery processes and that have been systematically evaluated within routine mental health care settings.

Inspired by the intervention mapping approach [28], input from various focus groups [27], a scientific literature review, and theory-related methods were combined to develop the innovative and device-friendly digital “STORY MINE” intervention [29]. The intervention is named after the eponymous STORY MINE research project, which stands for “Studying Topics of Recovery in Mental Health Oriented Narrative Experiences.” The intervention contains hopeful, purposefully collected narratives containing the following active ingredients expected to promote recovery: a diversity of narrators and experiences, authenticity, hopeful content, and a recovery phase between intermediate and full recovery. Narratives are offered in different lengths and modalities.

STORY MINE was explicitly designed as a theory-informed, transdiagnostic digital intervention, integrating curated recovery narratives within an existing eHealth platform used in routine mental health care. Our primary objective is to gain a comprehensive understanding of the feasibility, acceptability, and potential barriers to implementation through user experiences with STORY MINE. Second, we aim to gain insight into the potential impact on the outcome measures of mental health confidence, hope, self-stigma, and personal recovery and to inform a future randomized controlled trial (RCT).

## Methods

### Study Design

In this study, we evaluate the STORY MINE intervention by conducting a pilot RCT. The trial was designed as a low-risk feasibility study and was not intended to evaluate clinical efficacy. In line with feasibility study objectives, the study focused on process evaluation (feasibility, acceptability, and engagement) rather than hypothesis testing of effectiveness. The results of this study are reported in line with the CONSORT (Consolidated Standards for Reporting Trials; Checklist 1) extension of Pilot and Feasibility Trials 2016 [30].

### Participants

We opted for broad inclusion criteria due to the transdiagnostic relevance of RN content [31]: (1) receiving outpatient or polyclinic specialized mental treatment at the Lentis Psychiatric Institute; (2) aged more than 18 years old; and (3) sufficient understanding of the Dutch language. These criteria were selected to maximize ecological validity and reflect the intended transdiagnostic application of the intervention within routine mental health care.

Participants were excluded if they were currently or had recently (within 3 mo) been enrolled in one of 2 official recovery-oriented programs within our facility [32,33] in order to reduce contamination effects and isolate the contribution of the intervention, as both included peer support as a central component, and this could influence the same outcomes targeted by STORY MINE.

We aimed to recruit and randomize 24 eligible participants, taking a potential of 20% drop-out into account [34]. This sample size was considered sufficient to estimate feasibility parameters and inform future trial design.

### Ethical Considerations

The Medical Ethics Review Board of the University Medical Center Groningen (METc-UMCG) reviewed the study protocol and confirmed that the trial does not fall under the scope of the Dutch Medical Research Involving Human Subjects Act (reference number: METc2024/486). The study was conducted in accordance with relevant ethical guidelines and regulations [30]. Participants signed informed consent prior to inclusion in the study and received a €10 (US $12) voucher after study completion.

### Recruitment and Randomization

Participants were recruited from November 2024 to February 2025. Recruitment took place through physical presentations, messages on the organization’s intranet, distributing recruitment material to treatment locations (eg, flyers clinicians could hand out to clients and posters in waiting rooms), and a short recruitment video. Clinicians were actively asked to inform their clients about the STORY MINE pilot.

Using a self-enrollment approach, interested clients could sign up by email or a digital registration form, independently or with help from their clinician. No formal data were collected on nonresponse or refusal rates, as recruitment was open and voluntary.

Randomization was conducted and safeguarded by a senior researcher who was not involved in the STORY MINE trial. Participants were randomly assigned to the intervention group (ie, 12 wk access to the STORY MINE module, consisting of 12 recovery narratives complementary to care as usual; CAU) or to the waitlist control group (CAU), using block randomization with blocks of 4 participants (2:2). Information about the participants’ group allocation was provided to the research assistant after completion of the baseline questionnaire so the STORY MINE module could be activated for participants in the intervention group. After the postintervention questionnaire was completed, participants from the control group were also offered access to the STORY MINE module. Assessor bias was minimized, as study outcomes used registered usage data and self-report measures only. Participants were advised to contact their treating clinician or lived-experience professional if participation evoked distress. As STORY MINE was evaluated by the Medical Ethical Review Board as a low-risk adjunct to care as usual, no study-specific adverse event monitoring or formal distress-response protocol was implemented.

### The STORY MINE Intervention

The STORY MINE intervention is offered as a digital eHealth module within the secured client portal “Minddistrict” for service users at Lentis Psychiatric Institute, a large Dutch mental health facility with over 30,000 patient contacts per year. The STORY MINE module provides information about the research project, user instructions, and 12 recovery narratives from individuals who are in the final stages of recovery or are living well with their remaining mental health challenges (as categorized in INCRESE item 34 “Relationship with recovery”) [35], as these are expected to enhance hope and connectedness [12,27]. The narratives, purposively collected in video podcasts with semistructured interviews [29], offered hopeful content, insights, and advice to support others in their recovery process. To ensure the narratives would contain these active ingredients, narrators first participated in a preparatory meeting to familiarize them with the interview process. The goal was to create a safe and supportive environment and discuss the topics of focus to ensure a diverse and hopeful content of the narratives. Narrators self-assessed their recovery stage using the INCRESE-NL instrument (intermediate to full recovery) [36]. Each narrative is available in 3 formats: a video-podcast, a written summary, and an audio narration of the summary, which are displayed with a brief description of their content. The narrators represent a variation in ages and mental health experiences with psychosis, depression, autism, and trauma, among others (Multimedia Appendix 1 for the narrator characteristics). Service users follow a four-step process: (1) select a narrative, (2) engage in the modality of their choosing, (3) evaluate, and (4) return to narrative overview. Participants decide when and how many narratives they consume during the trial period.

### Sociodemographic and Clinical Characteristics

At baseline (T0) the following sociodemographic and clinical characteristics were digitally collected: age, biological sex, gender identity, sexual orientation, country of birth, type of lived experience and general diagnostics (eg, self-reported experiences of trauma, psychosis, autism; multiple answers allowed), illness duration, and relationship with recovery (ie, recovered, living well, making progress, surviving day-to-day, or rejects recovery).

### Feasibility

To assess feasibility, we recorded relevant events (eg, technical difficulties and reasons for delay or dropout) throughout the trial. Additionally, export data from the module were used to evaluate participant engagement with the module. Engagement was operationalized as the number of active login days and the number of narratives accessed within the intervention period. Furthermore, we inquired whether participants engaged with lived experience narratives outside STORY MINE during the trial and if engaging with STORY MINE narratives resulted in the desire to contact someone about their experiences with the narratives.

### Acceptability

The postintervention assessment (T1) in the intervention group included a set of digital quantitative items and open-ended questions about the experiences of using the STORY MINE eHealth module. These items were developed specifically for this study based on prior focus group findings and existing literature on recovery narratives and digital interventions [27-29] and were intended to capture perceived usefulness, emotional impact, connectedness, and usability.

Quantitative items assessed the module’s contribution to recovery and participants’ connectedness with narrators or narratives using 0%‐100% scales, modality preference (video, audio, text, or no preference), and user-friendliness using a 1‐10 rating scale (1=not user-friendly at all and 10=perfect user-friendliness). These quantitative items were followed by open-ended questions inviting participants to elaborate on their ratings. Furthermore, participants qualitatively described any positive or negative effects and provided suggestions for the overall improvement of the intervention. In addition, participants completed evaluations after each consumed narrative during the intervention. These included quantitative ratings of the narrative’s perceived contribution to recovery (ranging from 0%‐100%) and the used modality, followed by open-ended questions asking participants to elaborate on their rating.

### Outcome Measures

Both the T0 and T1 assessments included the following outcome measures, based on input from focus groups [27] and previous literature as part of the Intervention Mapping process [29]: the Mental Health Confidence Scale (MHCS: 16-item scale, range 16‐95, higher scores representing more mental health confidence) [37]; the Herth Hope Index (HHI: 12-item scale, range 12‐48, higher scores represent higher levels of hope) [38]; the Short Internalized Stigma of Mental Illness Scale (ISMI-10: 10-item scale, range 10‐40, higher scores represent greater self-stigma) [39]; the Mental Health Recovery Measure (MHRM: 30-item scale, range 30‐150, higher scores are more personal recovery) [40]. All outcome measures have sufficient validated psychometric properties, including good reliability (ie, Cronbach α ranging from approximately 0.70-0.95 across instruments) and construct validity in mental health populations.

### Data Analysis

Following the CONSORT extension to pilot randomized pilot and feasibility trials [30], descriptive analyses were reported. Analyses were performed following the intention-to-treat (ITT) principle. Standardized mean differences (SMDs) were calculated for continuous baseline characteristics to describe the magnitude of between-group differences at baseline. Participants’ characteristics and quantitative feasibility, acceptability, and outcome data were reported using standard methods for frequency rates, means, medians, percentages, and IQR.

Qualitative data from participants’ immediate evaluations of individual narratives were analyzed using qualitative descriptive content analysis [41,42]. Following familiarization with the data, the first author (ML) inductively developed categories by grouping similar responses. The finalized coding framework was subsequently applied to all responses, after which the resulting categories and their interpretation were discussed with the wider research team (JB, IvB, and SC) to ensure consistency and agreement. Representative quotations were then selected to illustrate each category. Responses to the other open-ended postintervention questions were read repeatedly and summarized descriptively by grouping recurring topics, which were illustrated using selected verbatim quotations. Because these questions were intended to contextualize the quantitative findings rather than generate new concepts, no formal coding framework was applied.

Changes between baseline and postintervention were examined by calculating the mean change scores, Cohen *d* effect sizes, and 95% CIs. An exploratory sensitivity analysis was performed excluding participants who experienced a mental health crisis during the intervention to assess the influence of these individual cases on the overall findings. As an exploratory analysis of engagement, Spearman rank correlations were calculated between the number of narratives consumed and changes in each outcome measure.

Given the exploratory nature of the study, no formal hypothesis testing was conducted, and results were interpreted descriptively. Data were analyzed in SPSS 29.0 (IBM Corp).

## Results

### Participant Characteristics

In total, 28 participants were included, who all completed both the T0 and T1 questionnaires (0% drop-out, Figure 1).

Baseline characteristics showed some differences between the intervention and control groups, as reflected by the absolute SMDs and their 95% CIs (Table 1). Overall, the sample distribution was skewed for sex (23/28, 82.1% women) and for type of self-reported psychiatric complaints (mood-related: 26/28, 92.9%; anxiety: 16/28, 57.1%; trauma or posttraumatic stress disorder [PTSD]: 17/28, 60.7%).

**Figure 1.**
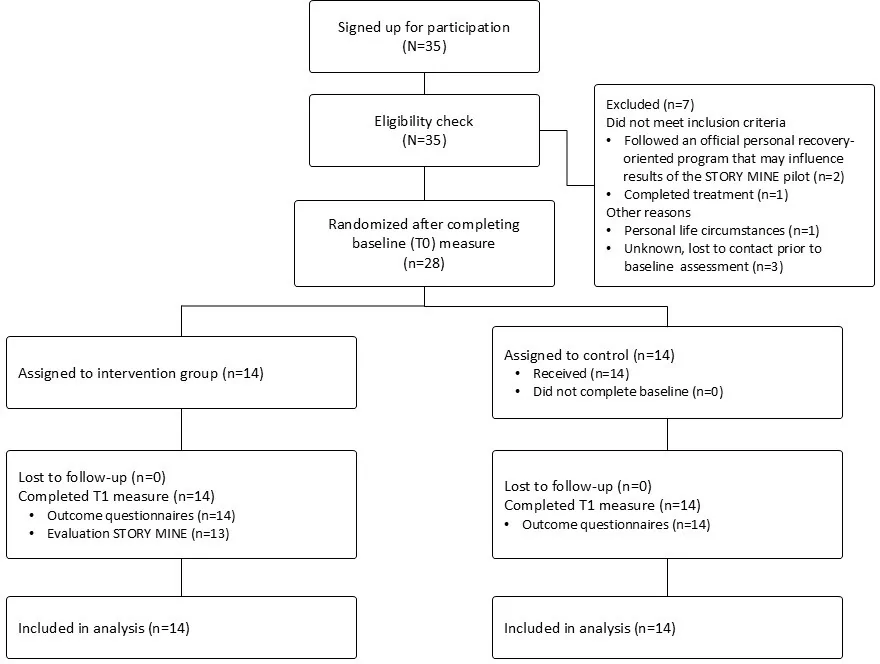
Participant flow diagram of a pilot feasibility RCT evaluating the STORY MINE digital recovery narrative intervention among adult outpatient service users receiving specialized mental health care at Lentis Psychiatric Institute (the Netherlands), recruited between November 2024 and February 2025. Note: One participant in the intervention group did not complete the STORY MINE evaluation due to incorrect group allocation at T1 and therefore did not receive the evaluation items. RCT: randomized controlled trial.

**Table 1.** Sociodemographic and clinical characteristics of participants at baseline (T0) in a pilot feasibility RCT of the STORY MINE digital recovery narrative intervention among adult outpatient service users receiving specialized mental health care at Lentis Psychiatric Institute (the Netherlands), 2024‐2025*.*

Characteristic	IG^a^ (n=14)	CG^b^ (n=14)	Total (N=28)	SMD^c^ (95% CI)
Age (y), mean (SD)	43.4 (13.01)	39.0 (10.17)	41.2 (11.68)	0.38 (−0.37 to 1.12)
Gender, n (%)				
Men	3 (21.4)	1 (7.1)	4 (14.3)	—^d^
Women	10 (71.4)	13 (92.9)	23 (82.1)	—
Transgender man	1 (7.1)	—	1(3.6)	—
Sexual orientation, n (%)				
Heterosexual	10 (71.4)	13 (92.9)	23 (82.1)	—
Non-heterosexual^e^	5 (35.7)	3 (21.4)	8 (28.6)	—
Country of birth, n (%)				
Netherlands	13 (92.9)	14 (100)	27 (96.4)	—
Other^f^	1 (7.1)	—	1 (3.6)	—
Self-reported psychiatric complaints, n (%)^g^				
Mood-related	13 (92.9)	13 (92.9)	26 (92.9)	—
Psychosis	4 (28.6)	—	4 (14.3)	—
Anxiety	8 (57.1)	8 (57.1)	16 (57.1)	—
Trauma / PTSD^h^	8 (57.1)	9 (64.2)	17 (60.7)	—
Autism	5 (35.7)	4 (28.6)	9 (32.1)	—
Addiction	4 (28.6)	1 (7.1)	5 (17.9)	—
Other	2 (14.2)	3 (21.4)	5 (17.9)	—
Illness duration (y), mean (SD)	18.9 (13.4)	12.1 (8.1)	15.5 (11.4)	0.61 (−0.15 to 1.37)
Recovery status T0, n (%)^i^				
Recovered	—	—	—	—
Living well	1 (7.1)	1 (7.1)	2 (7.1)	—
Making progress	9 (64.3)	7 (50.0)	16 (57.1)	—
Surviving day-to-day	4 (28.6)	5 (35.7)	9 (32.1)	—
MHCS^j^, mean (SD)	66.43 (8.59)	63.71 (11.05)	65.07 (9.81)	0.27 (−0.47 to 1.02)
HHI^k^, mean (SD)	32.64 (5.62)	29.21 (4.73)	30.93 (5.39)	0.66 (−0.10 to 1.42)
ISMI-10^l^, mean (SD)	22.57 (4.29)	22.00 (4.06)	22.29 (4.12)	0.14 (−0.60 to 0.88)
ISMI-10, mean (SD)	2.26 (0.43)	2.20 (0.41)	2.23 (0.41)	0.14 (−0.60 to 0.88)
MHRM^m^, mean (SD)	99.21 (14.58)	87.5 (14.90)	93.36 (15.65)	0.79 (0.02 to 1.57)

a IG: intervention group.

b CG: control group.

c SMD: absolute standardized mean difference, calculated as Cohen *d* using the pooled standard deviation.

d Not applicable.

e IG: bisexual (n=1), homosexual (n=1), queer (n=1), other/I don’t know yet (n=2); CG: bisexual (n=1), pansexual (n=1), I decline to answer (n=1).

f Croatia.

g For reasons of comorbidity, participants could report multiple psychiatric complaints.

h PTSD: posttraumatic stress disorder.

i One participant self-reported having autism and rejects the concept of recovery.

j MHCS: Mental Health Confidence Scale.

k HHI: Herth Hope Index.

l ISMI-10: a short version of the Internalized Stigma of Mental Illness Scale.

m MHRM: Mental Health Recovery Measure.

### Feasibility of the STORY MINE Module

Key feasibility outcomes and study process issues are summarized in Table 2. Quantitative analyses showed active engagement with the module varied, with participants logging in between 1 and 6 days during the 12-week intervention period and consuming between 0 and 7 narratives. Of the participants with zero engagement, 2 reported personal life circumstances that prevented use of the module.

**Table 2.** Feasibility outcomes and study process issues observed during the 12-week pilot feasibility randomized controlled trial of the STORY MINE digital recovery narrative intervention among adult outpatient service users receiving specialized mental health care (the Netherlands), 2024‐2025*.*

Domain and measure	Result
Engagement	
Active login days, mean (SD)	2.71 (1.49)
Narrative evaluations completed, mean (SD)	2.93 (2.50)
Participants consuming no narratives, n	4
Expressed wish to discuss experiences with others, n	2
Technical implementation and protocol adherence	
Initial login difficulties, n	≥2^a^
Delayed module activation, n	3
T1 completed>2 weeks late, n	7
Time between T0 and T1 (days), median (IQR)	96.5 (84.5‐102.3)
Duration of access (days), median (IQR)	86.0 (74.5‐96.5)
Confounding interventions, n	
Additional peer support activities (IG^b^)	4
Additional peer support activities (CG^c^)	4
Use of external recovery narratives (IG)	1
Use of external recovery narratives (CG)	1
Safety and adverse experiences, n	
Experiencing crisis	1
Experiencing negative effects	1

a Exact numbers were not systematically recorded.

b IG: intervention group.

c CG: control group.

Several practical challenges were encountered during the study. Minor login difficulties were reported by several participants and were generally resolved with support from the research team or mental health professionals. Delayed activation of the module resulted in a shorter intervention period where one participant received access for only 6 days. One participant was incorrectly allocated at T1 and therefore did not receive the feasibility and acceptability questionnaire.

Participants expressing a desire to discuss their experiences with the narratives did not reach out to have a conversation. One participant experienced a crisis during follow-up that appeared unrelated to the intervention, while another participant reported initially positive but later negative effects of the narratives. Suggestions for improvement in the qualitative analysis mainly concerned easier navigation within the module and reducing the number of emails, links, and logins required during the study.

### Acceptability of the STORY MINE Module

Quantitatively, the user-friendliness of the STORY MINE module was rated positively (mean 7.77, SD 2.52; with n=8/13 rating ≥8). Furthermore, participants indicated STORY MINE contributed at least somewhat to their recovery and that they experienced connectedness with one or more narrators (Table 3). Modality preference varied between participants. One of the 4 participants who listened to ≥2 narratives noticed whether the audio narrative was recorded with the narrator’s or researchers’ voice and expressed a preference for the narrator’s voice.

**Table 3.** Quantitative evaluation of the STORY MINE module. Feasibility and acceptability outcomes of the STORY MINE digital recovery narrative intervention in a pilot feasibility randomized controlled trial among adult outpatient service users in specialized mental health care at Lentis Psychiatric Institute (the Netherlands), assessed over a 12-week intervention period (2024‐2025).

Measure or question	Values
Feasibility measures (n=14)	
Amount of active logins (days), mean (SD; range)	2.71 (1.49; 1-6)
Evaluated narratives within module (n), mean (SD; range)	2.93 (2.50; 0-7)
Acceptability (n=13)	
Contribution to recovery (%), mean (SD; range)	34.8 (29.01; 0-80)
Positive experiences, yes (n)	5
Negative experiences, yes (n)	1
Experienced connectedness (%), mean (SD)	41.8 (27.46; 0-80)
Modality preference (n)	
Video	4
Audio	2
Text	6
Equal preference	1
User-friendliness rating, mean (SD; range)	7.77 (2.52; 1-10)

### Qualitative Findings From Open-Ended Responses

The qualitative evaluation of the postintervention open-ended questions (T1) is summarized in Table 4. Participants described positive experiences related to recognition, hope, and meaning-making, but some also reported a lack of contribution to recovery and occasional distress. Evaluations of individual narratives within the STORY MINE module immediately after consummation further showed that recognition of shared diagnoses, emotional struggles, and life themes enhanced the perceived value of specific narratives.

**Table 4.** Summary of qualitative findings from postintervention open-ended responses (T1) and immediate evaluations of individual recovery narratives collected during the 12-week pilot feasibility randomized controlled trial of the STORY MINE digital recovery narrative intervention among adult outpatient service users receiving specialized mental health care (the Netherlands), 2024‐2025^a^*.*

Source, categories, and findings	Illustrative quote
Postintervention responses (T1)	
Connectedness (n=7): shared experiences enhanced identification with narrators	“*Several stories had similarities to my own situation.*” [p10]
Recovery contribution (n=5): recognition, meaning-making, hope, practical tips, and reduced feelings of isolation	“*What I was able to do within the module has changed the way I see myself and my general problems in a positive way.*” [p10]
Lack of contribution (n=5): no fit with diagnosis or personal experiences (n=4), or narratives perceived as pleasant but without impact (n=1)	“*No, I can’t relate it to a similar situation.*” [p17]
Positive experiences (n=5): hope, inspiration, normalization, and reflection on personal goals	“*I found it helpful that she (ie, one of the narrators) said you have to be ready for treatment. Sometimes you’re just not there yet. In my case, I’ve also ignored my trauma for a long time and am now waiting for therapy to start addressing it.*” [p24]
Mixed emotional responses (n=1): initial comfort followed by distress and triggering memories	“*At first, it brought me comfort and support. It made me feel like I’m not on my own, but later it started to trigger me, bringing back painful memories and nightmares, and I began to feel down.*” [p35]
Suggestions for improvement (n=2): greater diversity and diagnosis-specific content	“*More people who ended up in psychiatric care as a result of a physical disability, including losing everything—their business, relationship, and contact with their children.”*
Immediate evaluations of individual narratives	
Recognition (*f*=17^b^): shared diagnoses, emotional struggles, and life themes enhanced perceived relevance	“*I recognized myself in [the story] and also in the struggle of blaming yourself, something I struggle with a lot. Maybe it’s something I can bring up in my psychotherapy.*” [p06]
Practical value and role modeling (*f*=19): tips, reminders, and aspirations to become a peer support worker	“*I could relate to him lying in bed with depression; I have that as well. Also, he is a peer support worker, which is something I might want to become as well.*” [p12]

a Participants could evaluate multiple narratives; therefore, frequencies of coded narrative evaluations may exceed the number of participants.

b *f*: frequency of narratives coded with the respective category.

### Preliminary Effects of STORY MINE

We found small negative effect sizes for mental health confidence, hope and internalized stigma, and a medium negative effect size for personal recovery. Means, change scores, and effect sizes for the quantitative outcome measures at baseline and postintervention are reported in Table 5. Negative change scores and effect sizes indicate a negative influence of the STORY MINE module on recovery outcomes, with the exception of self-stigma (ISMI). Two participants in the intervention group experienced a crisis during the intervention, thus substantially influencing the results in a negative direction in our ITT analysis. As an exploratory sensitivity analysis, excluding these 2 participants from the analyses resulted in noticeably smaller change scores (MHCS: mean_change_=0.25, SD 7.53; HHI: mean_change_=–0.50, SD 4.36; ISMI: mean_change_=0.42, SD 2.91; MHRM: mean_change_=−2.25, SD 15.93) and very small to small effect sizes (MHCS: Cohen *d*=0.16, 95% CI −0.61 to 0.93; HHI: Cohen *d*=−0.21, 95% CI −0.98 to 0.56; ISMI: Cohen *d*=−0.41, 95% CI −1.18 to 0.36; and MHRM: Cohen *d*=−0.27, 95% CI −1.04 to 0.50). These analyses should be interpreted as exploratory sensitivity analyses, illustrating the potential impact of individual cases on overall outcomes. When qualitative responses were considered alongside participant-level engagement and exploratory outcome changes, no consistent pattern emerged. One participant with marked deterioration reported emotional triggering; another did not relate being in crisis to the intervention, whereas others described predominantly positive experiences despite little or no quantitative improvement.

To explore the relationship between intervention engagement and outcome change, Spearman’s rank correlations were calculated between the number of narratives viewed and changes in each outcome measure. No clear associations were observed between intervention engagement and outcome changes (MHCS: ρ=−0.11; HHI: ρ=0.01; ISMI: ρ=0.02; and MHRM: ρ=−0.19).

**Table 5.** Outcome measures: means, change scores, and effect sizes by group. Changes in mental health confidence, hope, internalized stigma, and personal recovery from baseline (T0) to postintervention (T1) in a pilot feasibility randomized controlled trial of the STORY MINE digital recovery narrative intervention among adult outpatient service users receiving specialized mental health care at Lentis Psychiatric Institute (the Netherlands), 12-week follow-up (2024‐2025)^a,b^.

Outcome measures	Intervention group (n=14), mean (SD)			Control group (n=14), mean (SD)			Effect size, Cohen *d* (95% CI)
Pre	Post	Change	Pre	Post	Change	
MHCS↑^c^	66.43 (8.59)	61.36 (18.20)	–5.07 (15.80)	63.71 (11.05)	62.71 (13.49)	–1.00 (8.11)	–0.32 (–1.07 to 0.42)
HHI↑^d^	32.64 (5.62)	30.86 (7.19)	–1.79 (5.47)	29.21 (4.73)	29.57 (5.52)	0.36 (3.97)	–0.45 (–1.2 to 0.3)
ISMI-10↓^e^	22.57 (4.29)	23.21 (3.70)	0.64 (2.85)	22.00 (4.06)	23.57 (3.63)	–.57 (2.77)	–0.33 (–1.08 to 0.42)
MHRM↑^f^	99.21 (14.58)	91.71 (26.20)	–7.50 (20.91)	87.50 (14.90)	89.00 (14.90)	1.50 (11.77)	–0.53 (–1.28 to 0.22)

a ↑ Higher scores indicate improvement; ↓ lower scores indicate improvement; values are reported as mean (SD), except for the effect sizes; the negative effect sizes indicate a decline in recovery in the intervention group compared to control, except for internalized stigma.

b For internalized stigma, a positive change score indicates increased internalized stigma and thus a negative influence on recovery; there was less increase in internalized stigma in the intervention group compared to the control, although values in both groups indicate an increase in internalized stigma.

c MHCS: Mental Health Confidence Scale.

d HHI: Herth Hope Index.

e ISMI-10: Internalized Stigma of Mental Illness Scale.

f MHRM: Mental Health Recovery Measure.

## Discussion

### Principal Results

This pilot feasibility RCT aimed to evaluate the feasibility, acceptability, and preliminary outcomes of the STORY MINE intervention among service users receiving specialist mental health care. Overall, the findings indicate that STORY MINE is feasible to implement within routine care and is generally perceived as acceptable by participants. However, engagement with the intervention was low, experiences of recognition and relevance were mixed, and exploratory quantitative outcomes showed small to medium negative effect estimates, with uncertainty reflected in their 95% CIs. These findings reflect variability in participants’ experiences and outcomes, rather than a clear or consistent pattern across measures.

### Interpretation of Findings

Despite some minor logistical challenges, findings indicated that it was feasible to implement STORY MINE within routine mental health care, and participation in its evaluation was doable (0% dropout). However, half of the participants consumed fewer than 4 narratives (≤25% of the available content), and there were fewer than 3 registered login days on average, which equals less than once every 4 weeks. These results indicate low engagement of the module, which may partly explain the lack of positive outcomes. However, engagement was not associated with outcome changes in this pilot study, providing no clear indication of a dose-response relationship. This suggests that the amount of exposure alone may not determine recovery-related outcomes and highlights the need to consider how, rather than simply how much, participants engage with recovery narratives. This interpretation is consistent with findings from the NEON program, where improvements in quality of life were observed despite relatively modest engagement, suggesting that exposure to a greater number of narratives does not necessarily translate into greater benefit [13].

Low engagement should therefore not be interpreted solely as an indicator of limited feasibility or acceptability, as it may simply reflect variability in individual needs, experiences, and usage patterns. Measurable change in objective outcomes may take more time than the 12 weeks in this pilot study and may occur postintervention. Therefore, a longer follow-up measure is recommended.

Several factors could have contributed to the low engagement. Some participants may have felt sufficiently supported after engaging with only a few narratives, while others may have experienced competing life events or priorities that limited their use of the intervention. Some participants reported a lack of recognition (ie, identification with the narrative) or perceived relevance (ie, applicability to their own situation), resulting in disengagement. Other participants mentioned technical barriers such as login difficulties or problems with module navigation. Although some technical barriers were easily solved, others may be the result of the restrictions and characteristics of the Minddistrict platform that was used to deliver the intervention, such as navigation difficulties and repeated login procedures.

Also, the low-threshold and low-intensity nature of STORY MINE, since it was offered unguided, without regular prompts or instructions, may have led participants to prioritize their time differently. Moreover, variation in modality preferences (eg, text, audio, or video) suggests that acceptability and perceived credibility of the intervention may differ between individuals. Factors such as personal preferences, literacy, and contextual characteristics may influence both modality choice and engagement and should also be systematically examined in future research.

Among participants who consumed one or more narratives, qualitative responses were predominantly positive. They described experiencing diagnostic recognition (eg, psychosis and depression), transdiagnostic recognition (eg, bullying and self-blame), meaning-making, and helpful reminders. Neutral or negative feedback, such as perceived irrelevance and lack of recognition, was reported but occurred less often. These findings align with prior literature demonstrating that connection and identification are central mechanisms underlying the impact of recovery narratives [12,14], and that perceived similarity between narrator and recipient enhances engagement and learning from peer content [43-45]. Our qualitative findings also align with prior studies demonstrating that personal recovery is a transdiagnostic construct [31].

Although STORY MINE included an equal amount of male and female narratives, participants in the pilot study were predominantly female (23/28, 82.1%). Given that greater connectedness occurs when the narrator and the recipient share the same gender [12], a more balanced male and female ratio might have resulted in greater acceptability of the intervention.

Furthermore, our sample included a high proportion of participants with self-reported mood-related problems (92.9%) and PTSD (60.7%). Prior studies show that affective symptoms, in particular, strongly hinder personal recovery [46], possibly due to overlapping factors such as hopelessness, low self-esteem, stigma, and reduced quality of life [47-50]. PTSD has also been associated with increased severity of affective symptoms in psychosis [51], often identified as an important treatment target for recovery and posttraumatic growth [52-54]. For individuals with active PTSD symptoms, recovery narratives may therefore have limited effect and, in some cases, could trigger traumatic memories [55]. This supports the theory that individuals with PTSD are prone to overidentification [22] with negative experiences in a narrative, which makes it more difficult for them to identify with an alternative agentic life story [23]. Given that STORY MINE is a self-help intervention without supportive therapeutic guidance, these findings suggest that caution is warranted when including participants with unresolved trauma. In addition, the hopeful nature of the narratives may unintentionally induce upward social comparison in individuals who are currently struggling, in crisis, or in earlier stages of recovery [56]. Exposure to stories of people who are further along in their recovery journey may evoke feelings of inadequacy, hopelessness, or the perception of “not doing recovery well enough,” potentially contributing to emotional distress rather than hope.

Moreover, the mixed emotional responses observed in this study, including one report of distress triggered by narratives, are consistent with previous findings that recovery narratives can have both beneficial and adverse effects depending on the recipient and the circumstances of exposure [15,26]. Participants’ qualitative accounts reflected several mechanisms proposed in the NEON Impact Model, including emotional activation, upward social comparison, identification mismatch, and differences in timing or readiness for recovery [15,26]. These findings underscore that digital recovery narratives should not automatically be regarded as harmless interventions. Rather, their impact appears to depend on individual characteristics, recovery stage, and the ability to process emotional responses elicited by the narratives.

Personal recovery is dynamic and nonlinear [11]; what may appear as an initial negative experience due to increased self-awareness or emotional activation may later still induce positive change such as fostering awareness, help-seeking, and renewed motivation for recovery [15,26].

### Limitations

This study has several limitations that should be considered when interpreting the findings. Additionally, several baseline differences were present between the intervention and control groups, particularly for illness duration, HHI, and MHRM. Given the small sample size, such random baseline imbalances are common in pilot trials, and the observed between-group differences may partly reflect regression to the mean rather than a true intervention effect. The small sample size and pilot feasibility design further limit the ability to draw conclusions about effectiveness.

Engagement with the intervention was low, which may have reduced the potential to detect meaningful changes in outcomes. The use of self-enrollment recruitment may have resulted in a sample of relatively motivated participants, potentially limiting generalizability. External factors such as personal life events, crises, and participation in other peer support activities may have influenced outcomes independently of the intervention. Because no study-specific adverse event monitoring needed to be implemented, mental health crises were identified only retrospectively during data interpretation. Since distress or crises were retrospectively assessed, some adverse responses may have gone unnoticed, limiting our ability to assess this potential risk in real time, particularly in unguided digital interventions for vulnerable psychiatric populations. Follow-up time was brief (12 wk), limiting insight into potential long-term benefits and experiences. Finally, qualitative data were based on self-report and may be subject to recall or response bias.

### Considerations for Future Directions

The results of this pilot RCT led to 4 main points of consideration. Regarding engagement (1) and recovery-related effects, we found that even the most active participant consumed only half of the available narratives. This might suggest that an unguided self-help format may not provide sufficient support, which may partly explain both the low engagement and the mixed emotional experiences observed in this study. Guided use within STORY MINE, supported by mental health professionals or lived experience experts, should be considered to stimulate engagement and provide opportunities to discuss, normalize, and process emotional reactions arising from the narratives. Setting expectations prior to inclusion about the expected amount of engagement with the module (eg, evaluate >1 narrative per wk), combined with weekly email reminders, may further support sustained engagement.

### Emotional Safety

Emotional safety needs to be assured, since narratives can also negatively affect recipients [15]. In line with harm minimization strategies identified in previous research [17], we recommend that future implementation should not only include content warnings but also brief instructions on coping with emotional responses and a built-in function to report negative effects. Embedding safety mechanisms such as check-ins, clear support pathways, and timely access to professional or peer support may help to mitigate potential adverse effects. The option to contact a mental health care professional or lived experience expert should be clear and simplified.

### Technical Usability

Technical usability could be further optimized by improving the user interface, device optimization, and clear instructions. Also, a tutorial or walkthrough video for users may help make initial use of the eHealth module easier.

### Perceived Relevance and Recognition

Perceived relevance and recognition in the narrators and narratives should be further improved by increasing diversity in the collection [12,14,15]. The collection of narrators should match the clinical and demographic characteristics and experiences of its target population [57]. We used the “Inventory of Characteristics of Recovery Stories” (INCRESE) [35,36] to check which topics (section 7: Narrative content) are currently present in our collection and to identify gaps, for which additional narratives should be collected (Multimedia Appendix 2).

Future research should focus on evaluating a refined version of STORY MINE in a larger, adequately powered RCT, with stratification based on relevant clinical characteristics (eg, illness duration, recovery stage, crisis or not, and diagnostic categories such as mood-related disorders, autism, or psychosis) and include a longer follow-up.

### Conclusions

This pilot study indicates that STORY MINE is a feasible and acceptable digital intervention within specialized mental health care but highlights important challenges related to engagement, relevance, and emotional safety. The findings emphasize the complexity of implementing recovery narrative interventions in heterogeneous clinical populations. While qualitative findings suggest potentially beneficial mechanisms that warrant further investigation, the preliminary negative effect estimates observed in some outcomes suggest that unguided digital recovery narratives may not be equally beneficial for all service users, especially for people with traumatic experiences, and providing the option for guided support is recommended. A refined, safety-informed intervention and a fully powered RCT are needed to evaluate effectiveness and to determine for whom and under which conditions such interventions are most beneficial.

## Supplementary material

10.2196/83948 Multimedia Appendix 1 Narrator characteristics.

10.2196/83948 Multimedia Appendix 2 Narrative content represented in the STORY MINE collection.

10.2196/83948 Checklist 1 CONSORT 2010 checklist.

## Abbreviations

CONSORT: Consolidated Standards for Reporting Trials
HHI: Herth Hope Index
ISMI: Internalized Stigma of Mental Illness Scale
ISMI-10: Short version of the Internalized Stigma of Mental Illness Scale
MHCS: Mental Health Confidence Scale
MHRM: Mental Health Recovery Measure
PTSD: posttraumatic stress disorder
RCT: randomized controlled trial
