# Supplementary material for: The STORY MINE Digital Recovery Narrative Intervention for Personal Recovery in Mental Health Care: Pilot Randomized Controlled Trial
Source: JMIR Form Res. 2026 Sep 30;10:e83948. doi: 10.2196/83948 (PMC13626295; doi:10.2196/83948)
Supplement: Supplementary file 1 [file formative-v10-e83948-s001.docx]

**Appendix 2.** Narrator characteristics

*Demographic and clinical characteristics of the 12 narrators whose recovery narratives were included in the STORY MINE digital recovery narrative intervention.*

| **Characteristic** | **Total (*n*=12)** |
| --- | --- |
| Age, years M (SD) | 41.5 (10.57) |
| Gender, n (%) |  |
| Male | 7 (58.3%) |
| Female | 5 (41.7%) |
| Sexual orientation, n (%) |  |
| Heterosexual | 9 (75.0%) |
| Non-heterosexual^*^ | 5 (41.7%) |
| Country of birth, n (%) |  |
| Netherlands | 12 (100%) |
| Self-reported psychiatric complaints, n (%)** |  |
| Mood-related | 11 (91.7%) |
| Psychosis | 5 (41.7%) |
| Anxiety | 5 (41.7%) |
| Trauma / PTSD | 6 (50.0%) |
| Autism | 2 (16.7%) |
| Addiction | 1 (8.3%) |
| Other^***^ | 6 (50%) |
| Recovery status T0, n (%)**** |  |
| Recovered | 3 (25%) |
| Living well | 7 (58.3%) |
| Making progress | 3 (25.0%) |
| Surviving day-to-day | - |
| Rejects the concept of recovery | 1 (8.3%) |

*Note:* Participants could report multiple psychiatric complaints because of comorbidity. *Abbreviations*: SD = standard deviation.* asexual (n=1), bisexual (n=3), other/I don’t know yet (n=1); ** for reasons of comorbidity, participants could report multiple psychiatric complaints; *** AD(H)D (n=1), ADD (n=1), AD(H)D & Gilbert syndrome and possibly Ehlers-Danlos syndrome (n=1), a manic episode & episodes of depression and episodes of extensive alcohol consumption (n=1), depression & mourning and loss (n=1), I reject the concept of diagnosis / I describe my complaints differently, namely: An existential experience (n=1); **** two participants endorsed more than one recovery status recovered & rejects the concept of recovery (n=1), recovered & living well (n=1).
