# Supplementary material for: The STORY MINE Digital Recovery Narrative Intervention for Personal Recovery in Mental Health Care: Pilot Randomized Controlled Trial
Source: JMIR Form Res. 2026 Sep 30;10:e83948. doi: 10.2196/83948 (PMC13626295; doi:10.2196/83948)
Supplement: Supplementary file 2 [file formative-v10-e83948-s002.docx]

**Appendix 4.** Narrative content represented in the STORY MINE collection

Coded section 7 of the Inventory of Characteristics of Recovery Stories (INCRESE).

*Note*: Topics were coded as ‘Present’ when not presented as ‘Mainly Positive’ or ‘Mainly Negative’ (e.g. narrator is neutral, or has equally
positive and negative experiences).

| # | Item | Description | Present | Mainly positive | Mainly negative |
| --- | --- | --- | --- | --- | --- |
| 48 | Pregnancy/birth | Positive examples: wanted pregnancy, pregnancy as upward turning point  Negative examples: traumatic pregnancies/birth experiences, unwanted pregnancy, abortion, post-natal difficulties, difficulties with getting pregnant or ability to have children. |  |  | 10, 12 |
| 49 | Family | Positive example: emotional or practical support or care from family members, relationships with family as source of happiness or resilience  Negative examples: own or parents’ divorce, separation, relationship breakdown or ongoing conflict, death or loss in the family. | 2, 4, 5, 6, 11, 12 | 8 | 7, 9, 10 |
| 50 | Being in care | Positive or negative experiences of adoption, living in foster care or non-parental care. |  | 2 | 6 |
| 51 | Education | Positive examples: having positive opportunities for learning/training, engaging in self-education/self-help.  Negative examples: difficulties in school, school refusal, being excluded, not enjoying school being required to attend a course. | 2, 3, 4, 5, 6, 8, 11 | 9, 10, 12 |  |
| 52 | Friendships | Positive examples: support of friends.  Negative examples: breakdown of friendships, loneliness, social isolation, death of a loved one. | 1, 6, 7, 11 | 2, 3, 12 | 8 |
| 53 | Relationships | Positive examples: supportive partner, new relationship bringing hope.  Negative examples: difficulties or conflict with partner. | 10 | 3, 7, 11, 12 |  |
| 54 | Housing | Positive examples: having own home, home being a source of pride or sanctuary.  Negative examples: being vulnerably housed or homeless. | 2, 4, 6, 8, 10, 12 | 3 |  |
| 55 | Income | Positive examples: income enabling choices, improvement in income.  Negative examples: debt, financial difficulties, poverty, experience of the benefits system. |  |  |  |
| 56 | Work | Positive examples: providing meaning and purpose, developing positive identity.  Negative examples: burnout, work-related stress, unstable employment, unemployment, unwanted retirement. | 1, 7, 8, 10 | 2, 4, 5, 9, 11, 12 |  |
| 57 | Criminal justice system | Positive examples: prison providing security, a turning point or opportunities.  Negative examples: being arrested, being in prison or young offenders’ institution, family member being in prison. |  |  |  |
| 58 | Diagnosis | Positive examples: makes sense of a situation, is a relief, provides answers or sense of hope.  Negative examples: disagreement with diagnosis, feeling stigmatised, loss of hope. | 2, 4, 5, 6, 9, 10, 12 | 3 | 1 |
| 59 | Medication | Positive examples: felt better, relief, hope.  Negative examples: felt worse, did not help, was not voluntary, had unwanted side effects. | 5, 6, 8, 11, 12 | 2 |  |
| 60 | Relationship with mental health professional | Positive or negative experiences of relationships with e.g. community mental health team worker, crisis team, mental health nurse, psychiatrist, psychologist, social worker. | 10 | 1, 4, 5, 7, 9, 11 |  |
| 61 | Peer support | Positive or negative experiences of providing or receiving individual or group-based peer support. May be formal e.g. within mental health services or recovery colleges, or informal e.g. from support groups such as AA, Depression Alliance, Hearing Voices Network. | 6 | 2, 5, 8, 12 |  |
| 62 | Involuntary use of mental health services | Positive or negative experiences of experiences of e.g. being sectioned in hospital, compulsory medication, community treatment orders. |  |  |  |
| 63 | Hospitalisation | Positive or negative experiences of voluntary or involuntary psychiatric hospitalisation. | 1, 4, 11, 12 |  | 8 |
| 64 | Psychological services | Positive or negative experiences of experiences receiving or delivering talking therapies or counselling e.g. arts or creative, CBT, DBT, group therapy, psychotherapy, person-centred, psychoanalytic, psychodynamic, solution-focused. | 1, 6, 10 | 2, 3, 7, 8, 9 |  |
| 65 | Alternative therapies/healing | Positive or negative experiences of e.g. acupuncture, homeopathy, massage, meditation, Reiki, traditional Chinese medicine (TCM). |  |  |  |
| 66 | Being in natural environments | Positive or negative experiences of the natural environment or outdoor activities, green therapy. |  |  |  |
| 67 | Animals/pets | Positive or negative experiences of contact with animals including with therapy / emotional support dog, or care of a pet. |  |  |  |
| 68 | Community activities | Positive or negative experiences of engagement with community-based groups or organisations, whether or not mental health-related. | 6 | 11 |  |
| 69 | Hobbies/interests/creative activities | Positive or negative experiences of leisure activities, hobbies, recreational interests or creative activities e.g. art, crafts, music (listening or playing), performance, reading, dance, writing. | 4, 8, 11 | 1, 2, 6, 7, 9, 10, 12 |  |
| 70 | Physical activities | Positive examples: gaining or maintaining fitness, being involved in sports or other physical activities.  Negative examples: being unable to participate in such activities, participation leading to feeling worse. | 4 | 2, 6, 7 |  |
| 71 | Activism | Positive examples: participation in political or other activities to achieve change in a group, organisation, service or system.  Negative examples: participation in the above leading to e.g. burnout, being overwhelmed. | 8 | 6, 11, 12 |  |
| 72 | Spiritual/religious activities | Positive examples: attending a religious/spiritual community, group or place of worship, participating in spiritual practices e.g. prayer, meditation, retreats.  Negative examples: above activities as compulsory or contributing to mental health distress. |  | 2 | 11 |
| 73 | Stigma | Positive examples: rejecting or overcoming stigma or shame due to mental health issues or for other reasons, e.g. ethnicity, gender.  Negative examples: experiencing disapproval of others, or own shame due to mental health issues or other reasons. | 5 | 1, 3, 4, 12 | 8 |
| 74 | Caring responsibilities | Positive example: care of someone else giving a sense of pride.  Negative example: care of someone else worsening own mental health problems. |  | 2, 4 | 10 |
| 75 | Family experiences of mental health issues | Positive example: mental health issues of other family members leads to a shared sense of understanding.  Negative example: mental health issues of other family members has detrimental effect on narrator’s own mental health. |  |  | 10 |
| 76 | Diet/nutrition | Positive or negative experiences of diet or nutrition linked to improving, maintaining or worsening narrator’s mental health. |  |  |  |
| 77 | Volunteering | Positive or negative experiences of any unpaid work. | 6 | 3, 4, 12 |  |

**Not represented**: Income, Criminal justice system, Involuntary use of mental health services, Alternative therapies/healing, Being in natural environments, Animals/pets, Diet/nutrition

**Reference**:

Original INCRESE: Llewellyn-Beardsley J, Barbic S, Rennick-Egglestone S, et al. INCRESE: Development of an Inventory to Characterize Recorded Mental Health Recovery Narratives. *Journal of recovery in mental health*. 2020;3(2):25-44.

Dutch translation: Lases, M. N., Bruins, J., Ng, F., Rennick-Egglestone, S., Llewellyn-Beardsley, J., Slade, M., van Sambeek, N., Scheepers, F., van Balkom, I. D. C., & Castelein, S. (2024). Feasibility, validity and reliability of the Dutch translation of INCRESE (INCRESE-NL) inventory to characterize mental health recovery narratives. *Journal of Recovery in Mental Health*, *7*(1), 31–49.
